# Prospects for malaria control through manipulation of mosquito larval habitats and olfactory-mediated behavioural responses using plant-derived compounds

**DOI:** 10.1186/s13071-017-2122-8

**Published:** 2017-04-17

**Authors:** Jackson M. Muema, Joel L. Bargul, Sospeter N. Njeru, Joab O. Onyango, Susan S. Imbahale

**Affiliations:** 1 Department of Biochemistry, Jomo Kenyatta University of Agriculture and Technology, P.O. Box 62000-00200, Nairobi, Kenya; 2 Molecular Biology and Bioinformatics Unit, International Centre of Insect Physiology and Ecology, P.O. Box 30772-00100, Nairobi, Kenya; 3 Department of Medicine, Faculty of Health Sciences, Kisii University, P.O. Box 408-40200, Kisii, Kenya; 4 Present Address: Fritz Lipmann Institute (FLI) – Leibniz Institute of Aging Research, D-07745 Jena, Germany; 5 Department of Chemical Science and Technology, Technical University of Kenya, P.O. Box 52428-00200, Nairobi, Kenya; 6 Department of Applied and Technical Biology, Technical University of Kenya, P.O. Box 52428-00200, Nairobi, Kenya

**Keywords:** Malaria, Vector control, Anopheline mosquitoes, Plant-derived compounds, Larval habitat manipulation, Mosquito functional ecology, Integrated vector management

## Abstract

Malaria presents an overwhelming public health challenge, particularly in sub-Saharan Africa where vector favourable conditions and poverty prevail, potentiating the disease burden. Behavioural variability of malaria vectors poses a great challenge to existing vector control programmes with insecticide resistance already acquired to nearly all available chemical compounds. Thus, approaches incorporating plant-derived compounds to manipulate semiochemical-mediated behaviours through disruption of mosquito olfactory sensory system have considerably gained interests to interrupt malaria transmission cycle. The combination of push-pull methods and larval control have the potential to reduce malaria vector populations, thus minimising the risk of contracting malaria especially in resource-constrained communities where access to synthetic insecticides is a challenge. In this review, we have compiled information regarding the current status of knowledge on manipulation of larval ecology and chemical-mediated behaviour of adult mosquitoes with plant-derived compounds for controlling mosquito populations. Further, an update on the current advancements in technologies to improve longevity and efficiency of these compounds for field applications has been provided.

## Background

Ever since mosquitoes were discovered to transmit malaria parasites more than a century ago [1, 2], malaria remains a significant threat to human life, with major fatalities disproportionately inflicting children of less than 5 years and pregnant women [3]. Significant decline in global disease burden has been reported by various surveillance studies between 2000 and 2015 following intensive deployment of key interventions: indoor residual spraying (IRS), long-lasting insecticide treated nets (LLINs) and artemisinin-based combination therapies (ACTs) [4, 5]. However, the epidemiological burden of malaria persists in sub-Saharan Africa due to inevitable drug and insecticide-induced resistance [6–8]. This is reflected by residual transmission to the vulnerable groups that accounted for 92% of global deaths reported from African region in 2016 [3]. Residual transmission, which is characterised by shifts in vector feeding patterns, variation in species composition, insecticide-induced behavioural avoidance from IRS and LLINs, outdoor resting and increased outdoor parasite transmission, has emerged following the operational scale-up of these interventions [9–11]. It is postulated that the current tools could lead towards malaria elimination in various epidemiological settings by suppressing vectorial capacity [12]. Yet, the inexhaustive protection implicated by these interventions coupled with reduced susceptibility of malaria vectors to insecticides increases exposure risk and requires pivotal approaches to reduce the annual entomological inoculation rates (EIRs) to less than 1 [10, 13].

Since the inception of integrated vector management (IVM) model by Major Williams C. Grogas in the early 20th century and its adoption by world health organization (WHO) in 2004, significant research has been conducted in search of novel strategies to disrupt disease transmission cycle [14, 15]. These include genetic modification (sterile insect technique (SIT) and paratransgenesis), the use of microbial larvicides, transmission blocking interventions, mosquito behavioral modification, and the recent CRISPR Cas-9 mediated disruption of mosquito reproduction. However, one of the intriguing questions is: why is the fight against malaria still beyond the horizon? To address this fundamental question, Ferguson et al. pointed out that mosquito ecology stands out as the greatest obstacle to malaria elimination and eradication [16]. As many countries within the geographical malaria fringe strive to enter elimination phase, vector control, and the ultimate disruption of *Plasmodium falciparum* transmission cycle is faced with a multitude of challenges encompassing mosquito ecology, and a clear understanding is required to drive the envisioned goal to its realisation. According to Ferguson et al. the complexity of vector populations that evade control interventions [9], genetic variation of mosquito behaviour [17, 18], insecticide resistance [19, 20], and environmental changes [21] constitute the dynamic complex of mosquito ecology that favour propagation of parasite sporogonic stages. While major progress has been made to understand the ecology of malaria vectors, constraints in fully unravelling the interactions with other bio-factors within the ecosystem (such as competitors, predators, and preys in food web complexes) for amplification of malaria transmission risks present a great challenge towards malaria eradication [16]. Russell et al. proposed that the effective control of malaria could also be improved by approaches aimed at manipulating the adult vector behaviours that lead to outdoor transmission through avoidance of IRS-targeted killing [22].

In essence, mosquitoes require and acquire vital resources from the immediate environment to complete their life-cycle, and in turn, facilitate transmission of *P. falciparum* parasites to humans. These resources include aquatic breeding sites, carbohydrate sugar sources, blood hosts, and resting places which influence the capacity of mosquitoes to transmit malaria parasites. Despite the high ownership of LLINs and intensive IRS in malaria hotspots, these interventions have failed to break the transmission cycle sufficiently and to linearly push EIR to levels required for local elimination, a scenario creating malaria transmission heterogeneities [12, 23]. Entomological surveillance studies indicate that emergence of behaviorally-resistant and aggressive vectors that evade targeted killing of IRS and LLINs has contributed to high rates of outdoor *P. falciparum* transmission in different epidemiological regions [24, 25]. Imperatively, interruption of malaria transmission would require integrative interventions that limit mosquitoes from acquiring these resources. Therefore, in addition to the first line interventions (IRS and LLINs), larviciding and the mosquito olfactory system appears to be the targetable Achilles heel [26] that could be explored to considerably improve control of vector populations and malaria vector annual inoculation rates. There is a close association between vector density and entomological inoculation rates which are paramount parameters of vectorial capacity and malaria epidemiology [27]. Indeed, the vector-parasite-host interactions such as host seeking, blood feeding, parasite development and successful transmission to a natural mammalian host are fine-tuned by the mosquito larval ecology [28] in which olfactory system plays a primordial role [29]. Given the above, the quality of environment which the juvenile aquatic stages encounter during their development fundamentally influences the success of resultant adult mosquitoes as vectors [28, 30]. Consequently, suboptimal larval conditions have been reported to negatively implicate vector life history traits such as adult female body size, blood meal acquisition frequency and volume, reproductive viability and cycles, and vector longevity which directly impact vectorial capacity and competence [28, 31, 32].

### Functional ecology of malaria vectors

**Fig. 1:**
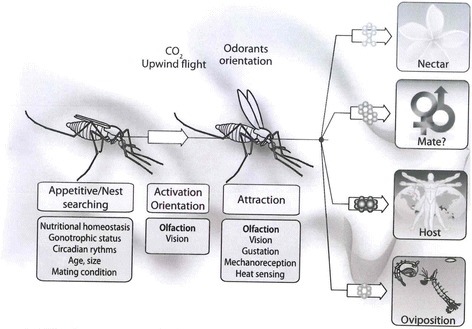
Mosquito olfactory-driven behavioral responses. Physiological status such as circadian-regulated appetitive stimulus or gonotrophic status activates olfaction in search of nutritional sources, mates and oviposition sites. On binding to odorant chemoreceptors and subsequent flight orientation, mosquitoes follow the source of the chemical cues for behavioral response depending on brain odor coding. Reproduced with permission of Wageningen Academic Publishers. Citation: Bohbot JD, et al. (2010) Molecular regulation of olfaction in mosquitoes. In: Takken W, Knols BGJ, editors. Olfaction in vector-host interactions. Wageningen, Netherlands: Wageningen Academic Publishers 2010; p. 17–38 [38]

Communication within and between insect species and subsequent interaction with natural environment depend chiefly on volatile organic compounds referred to as semiochemicals, which are chemical messengers selectively detected by the olfactory system from a sophisticated chemical ecology [33, 34]. Canonically, the olfactory system in insects not only provides a core link that coordinately mediates various behavioural and physiological responses to their external environment but also a guide towards their control [35, 36]. In mosquitoes, semiochemical cues characterise the functional ecology for oviposition site selection, copulation, host seeking, host selection and sugar foraging [29, 37, 38] (Fig. 1). For many years of insect research, olfaction has been a top priority in understanding chemical ecology with an evolutionary generalisation of various aspects based on *Drosophila* model [35, 39]. Based on this model, stimulant and inhibitory odorant compounds from natural environment are received by olfactory receptor neurones (ORNs) upon binding onto soluble odorant binding proteins (OBPs) expressed within the sensilla lymph of insect olfactory architecture [40, 41]. On solubilization, the odour complex is transported to odorant receptors (ORs) for detection [42] and subsequently, generates an action potential to the brain for odour decoding and behavioural response [43]. Over the last decade, several insect genome sequences including that of the malaria mosquito *Anopheles gambiae* [44] were annotated and released, tipping the comprehensive study of the olfactory system and design of robust control tools [45]. Some OBPs and ORs have been identified and characterised, with *An. gambiae* is having 276 G-protein coupled receptors (GPCRs) and 33 candidate OBPs that constitute the sensory pathway [46]. Comprehensive functional studies employing RNA*i*-mediated gene silencing, heterologous expression in an “empty neurone system” and electrophysiological assays have allowed elucidation of several volatile compounds that mosquitoes detect and respond to in varying degrees [47–49]. Several mosquito attractants and repellents have been extensively studied, in laboratory and semi-field settings, to unravel their roles in mediating distinct behavioural responses. For example, irrespective of the differential mosquito hosts’ attractiveness [50, 51], human skin emanations such as L-lactic acid and microbiota metabolites, exhaled breathe composition *viz* carbon dioxide (CO_2_), 1-Octen-3-ol and acetone constitute the principal mosquito attractants for host location and blood feeding [52, 53]. Binding of these chemical blends to the odorant receptors, AgGr22 on maxilla palp specific for CO_2_ and AgOR8 on antennal dendrites for 1-Octen-3-ol, stimulates chemosensation and flight-anemotactic behaviour for host seeking [29, 54]. On the other hand, geranyl acetate and citronellal from plant essential oils suppress sensitivity of specific receptors to host attractive cues mediating repellent effect against female *An. gambiae* vectors [48, 55].

Another important aspect of mosquito functional ecology with a great impact on vectorial capacity and malaria epidemiology is oviposition site selection, a critical factor in population dynamics. Evolutionarily, irrespective of the state of insecticide susceptibility, biting tendency and resting places, gravid female mosquitoes face the challenge of locating an appropriate oviposition site that guarantees progeny development and survival [56]. Following mating and bloodmeal acquisition, physiological changes that influence egg development and subsequent behaviour of searching an oviposition site ensue [57]. In addition to visual and tactile cues, the gravid females use to a larger extent olfactory signals to discriminatively select potential breeding sites [58–60]. A considerable number of field studies have reported the role of volatile organic semiochemicals emanating from aquatic habitats to attract or repel mosquitoes in a push-pull manner [61–65]. These semiochemicals interact with female chemoreceptors for cognition before egg laying process [60]. For example, in the event of characterising odour coding in mosquitoes, Carey et al. [48] and Rinker et al. [66] demonstrated that perception of oviposition cues such as 3-methylindole, indole, 2-propylphenol, and 4-methylcyclohexanol by female chemoreceptors could be presumably responsible for inducing egg laying. Importantly, volatile organic compounds produced by habitat-associated bacteria such as fatty acids, aromatic amino acids (L-tyrosine, L-phenylalanine and L-tryptophan), and carbohydrates show attraction to *Aedes aegypti* and *Culex pipiens* [67, 68]. While efforts to identify the key compounds mediating attraction and selective behaviour in mosquitoes had been made decades ago for *Aedes* and *Culex* [69], those responsive to anophelines lagged behind [70]. It was until recently when Lindh et al. [71] reported cedrol, a fermentation metabolite produced by fungi found on rhizomes of *Cyperus rotundus* grass, as a strong oviposition attractant for gravid *An. gambiae* (*s.s.*) mosquitoes that aroused interests to characterise anopheline oviposition ecology. In course, Wondwosen et al. [72] showed that volatiles from various rice cultivars strongly attracts gravid *An. arabiensis* females. In this study, using electrophysiology and chemical analyses, the authors demonstrated that headspace rice volatiles rich in β-caryophyllene, decanal, sulcatone (6-methyl-5-hepten-2-one) and limonene elicited antennal responses that on evaluation with BioGent (BG) sentinel traps stimulated long range oviposition site seeking behaviour. Elsewhere, Eneh et al. [73] reported that *p*-cresol, a strong oviposition attractant of *Aedes* and *Culex* from Bermuda grass (*Cynodon dactylon*) hay infusion, elicited avoidance response to gravid *An. gambiae* (*s.s.*). Cumulatively, these studies open avenues for the search of other compounds acting together to mediate the responses that could aid in developing not only general but also species-specific oviposition deterrents or attractive lethal ovitraps (ALOTs). Studies characterising the bio-physicochemical parameters of larval habitats show that habitat selection by female mosquitoes is species-specific and anopheline mosquitoes prefer shallow, temporary, less turbid, and open sunlit water bodies with adequate food resources and absence of predators [74–76]. However, in some extreme cases, these larvae have been reported to colonise polluted urban waters, a phenomenon depicting some form of adaptive divergence [77]. Also, the observed proximity of anopheline larval habitats to human dwellings was suggested to be an evolutionary strategy of mosquitoes to conserve energy for host seeking [78]. Therefore, interventions aimed at manipulating or disrupting the sensory signals in mosquitoes could lead to abrogation of olfactory-driven behaviours that contribute to malaria transmission [45, 79].

Indeed, approaches that target the behavioural attributes of adult mosquito vectors and their juvenile aquatic stages using chemical insecticides have been shown to substantially reduce mosquito populations [80–82]. However, the negative implications such as chemical pollution, loss of biodiversity, and the emergence of resistance associated with their application call for urgent interventions that safeguard environmental health as well as effectively reduce the risk of malaria transmission [83]. Over time, a paradigm shift in vector control has been experienced as the application of natural products especially from plants continues to be appreciated [84–88]. Hence, prospecting for bioactive chemistries from natural sources forms a basis for developing eco-friendly insecticides with less impact on biodiversity [89, 90]. Various approaches ranging from basic evaluation to complex molecular and *in silico* ligand docking studies, in search of inexpensive, safe, and effective classes of compounds have been deployed as pathways for developing new mosquito control agents [91, 92]. Although this does not guarantee 100% resistance-resilience, we feel that motivation for studying plant-based insecticides *vis-à-vis* their synthetic counterparts originate from previous reports showing few or no cases of resistance development to natural compounds derived from plants. In nature, plants constitutively and inductively synthesise a myriad of bioactive allelochemicals such as alkaloids, terpenoids, flavonoids, coumarins, glycosides, steroids, tannins, protease inhibitors, phenolics and growth regulators to counteract environmental stress effects and herbivory attacks [93, 94]. These harmful compounds have the potential of controlling both medically and veterinary important disease-transmitting insect vectors owing to their chemistry and structural characteristics that alter the normal physiology of insects, thus reducing their fitness and performance [95].

This review mainly focuses on the manipulation of larval habitats and chemical-mediated behaviour of adult mosquitoes using plant-based chemicals for reduced risk of malaria transmission. On application, plant-derived compounds could alter the immediate chemical ecology of mosquitoes disrupting the olfactory-mediated location of vital resources required for completing their life-cycle and transmitting malaria parasites. Importantly, we demonstrate how these compounds could be incorporated into IVM programmes for mosquito control.

### Push-pull technology for control of adult mosquitoes

The push-pull approach is not a new terminology in the context of pest management. The technique was first developed by Australian researchers, Pyke et al. [96] to manipulate the distribution of cotton pests of *Helicoverpa *spp. By then, scientific information on behavioural manipulation for controlling nuisance insects was rudimentary. A comprehensive review compiled by Foster & Harris [97] advanced and provided a clear understanding of behavioural manipulation for pest management that involves the use of stimuli to activate or inhibit a behaviour thereby changing its expression. From this knowledge, a push-pull approach for controlling mosquito vector populations by manipulating the vector behaviour and their relative spatiotemporal distribution for trapping and subsequent killing of the trapped insect vectors was conceptualised and adapted about 20 years later [98]. This technology has been successfully applied for controlling populations of mosquitoes [98], tsetse flies [99], as well as stem borers and *Striga* weeds from maize plantations [100–102]. In this context, repellents and baits are integratively deployed for driving the mosquitoes away from their vertebrate hosts and luring them towards the trap. For instance, the combinatorial use of a trap baited with an attractant blend that simulated human sweat and a microencapsulated synthetic repellent (δ-undecalactone) in malaria endemic region of western Kenya reduced mosquito house entry by more than 50% and high numbers of outdoor flying mosquitoes captured [103]. Although the use of repellents may deflect mosquitoes from repellent users to non-users [104], the dual deployment of repellents and attractive traps in push-pull systems would reduce vector densities and EIRs experienced by unprotected people in epidemiological settings by substantial fold [103]. Under such conditions, mosquito olfactory system acts as the possible target to deprive acquisition of resources from the host and environment. Carey & Carlson [45] pointed out that utilisation of cheap, stable and less hazardous compounds that either stimulate or inhibit mosquito odorant receptors, gustatory receptors and ionotropic receptors could lead to the development of effective, eco-friendly vector control tools that overcome the challenges faced by the current mosquito control strategies. Thus, in an attempt to implement this robust system at remote settings, the design of low-tech and non-power dependent traps that deploy natural products is highly encouraged to reduce human-biting mosquito populations. Combined use of plant-derived compounds with repellent and luring characteristics has the potential of designing such novel push-pull systems. For example, nepetalactone from *Nepeta cataria* (catnip) essential oil [105] and linalool oxide [106] are potent plant-based compounds that could be utilised in the “push” and “pull” effects, respectively. Inspirations from this technique have observed considerable success in the small scale mass trapping of mosquitoes and are currently under field expansion trials in Kenya and Tanzania [103, 107]. To discuss how plant-derived compounds could be applicable in the push-pull approach, we have split it into plant-derived insect repellents and ‘attract and kill’ phenomenon using toxic sugar baits.

### Plant-derived insect repellents

An insect repellent is presumably a compound that acts singly or in a cocktail of others to successfully deter a nuisance insect from locating the source of attractive host stimuli. Based on the induced insect behaviour, repellents can be broadly classified into; stimuli-irritants, odour masking and feeding deterrents [108]. In this context of mosquitoes, by sensing or coming into contact with the compound, stimuli-irritants induce behavioural avoidance from the source of the chemical. Odour masking compounds reduce the abundance of host attractive cues while feeding deterrents interfere with bloodmeal and nectar sugar acquisition. To mediate repellent effect, the sensation of these aversive compounds to mosquito sensilla may activate specific insect ORs, block firing of neuron currents or disrupt behavioural responses [109–111]. It remains a hot debate within the malaria community on whether to repel or kill mosquitoes [112]. Nevertheless, the aim of either topical or spatial application of repellents is to disrupt the mosquito olfactory signalling and subsequent host-seeking behaviour.

Before the advent of synthetic chemical repellents, man used and still uses plants with repellent characteristics to drive away mosquitoes with or without the knowledge of their efficacy, mode of action and their safety [113]. As early as 1901, botanical derivatives such as essential oils of citronella (*Cymbopogon* spp), neem (*Azadirachta indica*) and lemon eucalyptus (*Eucalyptus maculata*) were used in ancient Greece, Egypt, China, India and even northern America to ward off biting insects and protect crops against destructive pests [114]. These botanicals are effective even up-to-date, however, due to their high volatilization, their reliability dropped in 1953 for synthetic repellent DEET (N, N-diethyl-3-methylbenzamide) which was adopted as the gold standard mosquito repellent in the United States [115]. DEET is effective against bites of most disease-transmitting vectors offering up to 99.9% personal protection for the long residual period. Although the mechanisms of action of DEET have been debatable for several years, initial studies hypothesised that DEET masks lactic acid on the human skin thus reducing attraction to biting mosquitoes [116]. However, though controversy still exists, molecular and functional studies disputed this notion and showed that DEET selectively inhibits specific insect ORs by blocking electrophysiological signals of sensory neurones to attractive stimuli [109, 117, 118]. These findings were seconded by De Gennaro et al. [119] who showed that mosquitoes with non-functional OR complexes were only responsive to contact with DEET. Findings from Bohbot & Dickens [118] suggested that the structurally diverse repellent compounds including DEET, IR3535 (3-(N-acetyl-N-butyl) amino propionic acid ethyl ester), KBR 3023 or Picaridin (2-(2-hydroxyethyl)-1-methylpropylester) and MR08 (menthol propylene glycol carbonate) modulate the function of mosquito odorant receptors reducing vector-host contacts. Despite the excellent efficacy of DEET against mosquitoes, its use has been associated with various challenges. Its cost ineffectiveness and chronic human toxic effects [120] coupled with recent reports of resistance [121–123] compromise user reliability and human beings seem to have diverted preference to cheap, safe, eco-friendly and effective natural products of plant origin [90]. A review by Maia & Moore highlighted some adverse side effects such as dermatitis sensation that resulted from the application of plant essential oils, thus in their view, plant-derived compounds may not be necessarily safer than synthetic insecticide DEET [86]. However, the documented chronic side effects of DEET in children [120] surpass those implicated by plant derivatives.

Studies continue to report plants as potential sources of effective insecticides and interest on studies of plant-derived repellents renewed [86, 114, 124, 125]. For instance, pyrethroids used to impregnate LLINs were initially sourced from *Chrysanthemum* plant extracts (pyrethrum) in Kenya [126]. With the close interactions of humans and nuisance mosquitoes that bay for blood, local communities in malaria endemic regions have devised cheap means of trying to drive these organisms out of their reach by using plants. In the African region, ethnobotanical knowledge has been immensely deployed to identify plants with repellent characteristics used by local communities to drive away mosquitoes from human dwellings as a preliminary source of mosquito control agents [127–131]. These surveillance studies show that bruising, burning or smouldering of the repellent plant parts, planting repellent plants around homesteads, and topical application of plant-derived oil formulations on the skin and garments are common local practices for keeping mosquitoes away from human hosts. A well-documented example was where Seyoum et al. reported that natives from western Kenya drove away *An. gambiae* (*s.s.*) mosquitoes from their huts *via* the direct burning of *Lantana camara, Azadirachta indica*, *Lippia ukambensis*, *Tagetes minuta*, and *Ocimum americana* [132]. It has been suggested that the smoke produced by burning these plants masks human kairomones and convention currents used by mosquitoes for host seeking [133]. Also, the smoke lowers relative humidity making mosquitoes vulnerable to desiccation and reducing sensory input as mosquito receptors respond well in the presence of moisture [133]. Though this may appear primitive, this local method of reducing vector-human contacts forms the basis of today’s formulations against nuisance mosquitoes, and thus ethnobotanical knowledge has played a significant role in the search of natural products with repellent properties [88]. Plant-derived compounds applied on human host skin surface, or space spraying interferes with mosquito host-seeking and blood feeding process [86]. Given the fact that mosquitoes detect and respond to host volatiles, it is conceivable that reduction of the relative abundance of each chemical cue detected using repellents during sampling process would significantly minimise overall attraction.

**Fig. 2:**
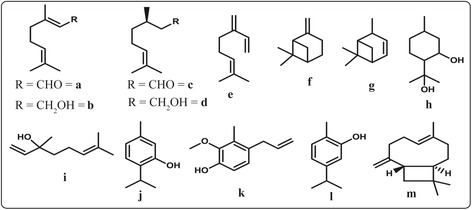
Plant-derived insect repellent compounds: **a** Citral, **b** Geraniol, **c** Citronellal, **d** Citronellol **e** Myrcene, **f** α-pinene, **g** β-pinene, **h** *p*-menthane-3,8-diol (PMD), **i** linalool, **j** Thymol, **k** Eugenol, **l** Carvacrol, and **m** Caryophyllene

The most plant-derived compounds reported to possess mosquito repellent effects include citral, geraniol, citronellal, citronellol, myrcene, α-pinene, β-pinene, *p*-menthane-3,8-diol (PMD), linalool, thymol, eugenol, carvacrol and caryophyllene [86, 124, 134] (Fig. 2). These natural compounds which potentiate excited-repellent effects are flavours and fragrances of plant essential oils mainly categorised as monoterpenes (acyclic and cyclic), sesquiterpenes and aliphatic compounds (alkanes, alkenes, ketones, aldehydes, acids and alcohols) [90]. Among these constituents, terpenes (monoterpenes and sesquiterpenes) have been reported to be as effective as DEET in intoxicating insects when topically applied or sprayed in space [90].

In fact, the US Centers for Disease Control and Prevention (CDC) recommends the use of repellents against mosquito-borne diseases for travellers and military [135, 136]. For instance, potent plant-derived repellents such as *p*-methane-3,8-diol (PMD) (from *Eucalyptus* spp.) and citronella oil (from *Cymbopogon* spp.) are fully registered and recommended for topical use because of their protection efficacy of over 95% against mosquitoes [137, 138]. This report is supported by field studies, conducted in Ghana, where subjects wearing PMD- and citronella-treated garments were significantly protected from mosquito bites [115]. Furthermore, topical application of PMD repellent on forearms by subjects in South Africa offered long-term 90–100% (for 5–6 h) protection against *An. arabiensis* mosquito bites which were equally effective as the commercial standard, DEET [139].

Laboratory evaluations by Deletre et al. [140, 141] highlighted strong repellency and electrophysiological responses from pure aldehydes of essential oil relative to the constituent monoterpenes against *An. gambiae* (*s.s.*), a potential observation that warranted consideration of these plant-derived compounds to replace pyrethroids in impregnation of bed nets and window curtains. The tested essential oils were derived from plants: *Thymus vulgaris*, *Cymbopogon winterianus*, *Cuminum cyminum*, and *Cinnamomum zeylanicum*. It was observed that the activity of the individual major constituents could not match with that of the parent oils suggesting coherent interactions of the individual compounds to potentiate bioactivity. It is worth noting at this point that strong electrophysiological response elicited by compounds does not necessarily correlate to their repellent characteristics. In the arms-in-cage assay, Omolo et al. [55] tested the repellent activity of essential oils extracted from selected Kenyan plants; *Conyza newii, Plectranthus marrubioides*, *Lippia javanica*, *Lippia ukambensis*, *Tetradenia riparia*, *Iboza multiflora* and *Tarchonanthus camphoratus*. Findings from this study showed that application of the essential oils on arms of volunteers strongly elicited repellent efficacy of 79–100% protection against *An. gambiae* (*s.s.*) mosquitoes are deterring host blood feeding. Major constituents of the essential oils eliciting repellent effects were identified as perillyl alcohol, *cis*-verbenol, *cis*-carveol, geraniol, citronellal, perillaldehyde, caryophyllene oxide and a sesquiterpene alcohol. They further tested the repellent activities of the most abundant compounds within synthetic blend formulations for different plant essential oils. Four formulations of *C. newii* - perillaldehyde, perillyl alcohol, 1,8-cineole, limonene [29:4:10:7]; *T. riparia* - fenchone, limonene, 1,8-cineole, [64:2:1.5]; *T. camphoratus* - camphene, α-pinene, α-fenchyl alcohol, 1,8-cineole, α-terpeneol, *p*-cymene [17:17:15:7:4:3]; and *L. javanica* - limonene oxide, *cis*-verbenol, verbenone, linalool, limonene, α-terpeneol [39:11:6:3:2.5:2] were found highly comparable to the activity of individual crude essential oil. In another study, Wanzala & Ogoma [142] evaluated the repellency efficacy of essential oil extracted from *Tagetes minuta* against *An. arabiensis* females. The essential oil rich in ocimene, tagetones, dihydrotagetone, ocimenones, piperitenones, 3,9-epoxy-*p*-metha-1,8(10)diene, β-caryophyllene, bicyclogermacrene, and AR-turmerone significantly deterred host seeking and biting by female mosquitoes relative to control subjects who applied vaseline petroleum jelly on their arms. This protective effect of plant-based repellents has been extended to planting repellent plants around homesteads and thermal expulsions that reduce the rates of mosquito entry into the houses [86, 132, 143, 144]. Repellent efficacy of plant-derived compounds has been summarised in Table 1.

**Table 1:** A summary of some repellent plant derivatives against anopheline mosquitoes

Plant	Major repellent compounds	Mode of testing	Repellency efficacy	Study type	Reference
*Conyza newii*	Perillyl alcohol, perillaldehyde, geraniol	Topical application	100% protection	Laboratory study	[55]
*Mkilua fragrans*	Linalool, camphor, 4-isopropylbenzenemethanol, carvone, caryophyllene oxide	Topical application	RC_50_ 9.21 × 10^-5^ mg cm^-2^	Laboratory study	[125]
*Endostemon tereticaulis*	Terpene-4-ol, fenchone, γ-terpinene, terpinolene	Topical application	RC_50_ 1.52 × 10^-5^ mg cm^-2^	Laboratory study	[125]
*Ocimum fischeri*	Eugenol, terpinolene, β-myrcene	Topical application	RC_50_ 0.67 × 10^-5^ mg cm^-2^	Laboratory study	[125]
*Ocimum forskolei*	Fenchone, camphor,α-pinene, β-myrcene	Topical application	RC_50_ 1 × 10^-5^ mg cm^-2^	Laboratory study	[125]
*Plectranthus longipes*	Carvacrol, caryophyllene oxide, terpene-4-ol, β-myrcene, γ-terpinene, α-terpinene	Topical application	RC_50_ 1.93 × 10^-5^ mg cm^-2^	Laboratory study	[125]
*Croton pseudopulchellus*	Linalool, caryophyllene oxide,γ-terpinene, 1-methylpyrrole	Topical application	RC_50_ 3.74 × 10^-5^ mg cm^-2^	Laboratory study	[125]
*Nepeta cataria*	Caryophyllene, nepetalactone	Topical application	RC_50_ 0.081-0.091 mg cm^-2^	Laboratory study	[105]
*Lantana camara*	Caryophyllene	Direct burning	27.22–43% protection	Field study	[132]
*Thymus vulgaris*	α-terpinene, thymol, linalool, geraniol, carvacrol, *p*-cymene	Topical application	> 80% protection	Laboratory study	[141]
*Azadirachta indica*	Azadirachtin, saponins	Direct burning	25–94% protection	Field study	[132]
*Corymbia citriodora*	*p*-menthane-3,8-diol, citronella, citronellol, geraniol, limonene, isopulegol, δ-pinene	Topical application	48–100% protection	Field study	[139]
*Plectranthus marrubioides*	Camphor, 1,8-cineole, *p*-cymene,aterpenene, fenchone, isocaryophyllene	Topical application	100% protection	Laboratory study	[55]
*Tarchonanthum camphoratus*	Camphene, α-pinene, α-fenchyl alcohol, 1,8-cineole, α-terpeneol, *p*-cymene	Topical application	99% protection	Laboratory study	[55]
*Tetradenia riparia*	Fenchone, limonene, 1,8-cineole	Topical application	80% protection	Laboratory study	[55]
*Lippia ukambensis*	Myrcene, linalool, α-pinene, eucalyptol, camphor, camphene, 1,8-cineole	Topical application	84% protection	Laboratory study	[55]
*Lippia javanica*	Allopurinol, camphor, limonene, verbenone, α-terpeneol, limonene oxide, *cis*-verbenol, linanool, α-terpeneol	Topical application	90% protection	Laboratory study	[55]
*Tagetes minuta*	Ocimene, dihydrotagetone, tagetones, ocimenones, piperitenone, 3,9-epoxy-p-metha-1,8(10)diene, β-caryophyllene, bicyclogermacrene, AR-turmerone	Topical application	> 80% protection	Laboratory and semi-field studies	[142]

### Mechanisms of action of plant-derived insect repellents

Until today, the modes of action of most plant-derived repellent compounds are still unclear although neurotoxic effects involving gamma aminobutyric acid (GABA), octopamine synapses, inhibition of acetyl cholinesterases and regulation of ion channels have been characterised [90]. Binding of thymol to GABA receptors blocks the GABA-gated chloride channels on postsynaptic neurone membranes resulting in CNS hyper-excitations, convulsions and death [145]. Eugenol activates octopaminergic receptors reducing production levels of cyclic AMP (cAMP) [146]. Also, eugenol has been reported to increase the intracellular levels of calcium ions, thus inducing toxicity by mimicking the action of octopamine [146]. Other essential oil constituents inhibit acetyl cholinesterase (AchE) resulting in ataxia, either by irreversible inhibitory effect or reversible competition for the enzyme’s active site [147]. Geraniol and linalool reversibly compete with hydrophobic functional groups of AchE’s active site. Also, linalool was shown to inhibit neuronal electrical activity by inducing a reduction in amplitude of action potential and subsequent decrease in post hyperpolarization phase and firing frequency of action potentials [90]. Using *Drosophila*, Kwon et al., [148] demonstrated that citronellal interacts with transient receptor potential channel (TRPA1) modulating the Ca^2+^-dependent activation of potassium channel, but in *An. gambiae* TRPA1 is directly activated by citronellal. Loss of Ca^2+^-activated K^+^ channel resulted in impaired citranellal-elicited avoidance and increased the frequency of action potential in olfactory receptor neurones. In another study, plant essential oils from *Verbenaceae*, *Lamiaceae*, *Asteraceae* and *Rivularaceae* families were reported to inhibit mosquito odorant degrading enzymes of cytochrome P450 family on a metabolic standpoint [149]. Taken together, these compounds disrupt various insect cellular activities and biological processes conferring repellent or toxicity effect. The repellent efficacy of plant essential oils varies significantly according to the phytochemical profile of the plant extract and the target insect. On the other hand, toxicity is influenced by the chemical composition of the essential oil, which depends on the source, season and ecological settings, extraction method, time of extraction and plant part used for extraction [150].

Other plant compounds elicit oviposition deterrence effects to gravid female mosquitoes by rendering the site unfavourable for egg laying. For instance, dual choice experiments performed using essential oils of *Ocimum kilimandscharicum,* and *Ocimum suave* deterred gravid *An. gambiae* (*s.s.*) mosquitoes from laying eggs as shown by reduced egg count about controls [151]. (*E*)-caryophyllene and α-humulene from the essential oil of *Commiphora leptophloeos* have shown oviposition deterrence to *Aedes* mosquitoes, suggesting their potential to deter anopheline mosquitoes as well [152].

### Attract and kill phenomenon using attractive toxic sugar baits

Mosquitoes supplement nutritional requirements by foraging nectar sources to provide energy for flight, longevity and enhance fecundity [153, 154]. Hien et al. [155] showed that plant sugar sources differentially influence infection prevalence and intensity, and hence natural sugar sources present a great threat to control of malaria by enhancing the survival and fecundity of mosquito vectors as well as development of *P. falciparum* [156, 157]. This finding is supported by Nyasembe et al. who demonstrated that infection with *P. falciparum* stimulates urge of nectar sugar uptake by female mosquitoes [158]. Indeed, studies utilising behavioural response assays performed in dual-response olfactometer and coupled gas chromatography electroantennogram detectors (GC-EADs) have shown that anopheline mosquitoes discriminatively prefer certain plant odours for foraging [159], providing a basis for developing mosquito odor-baited traps using plant-based lures [160]. Extensive behavioural and chemical ecology studies have recently come up with attractive toxic sugar bait (ATSB) method that kills mosquitoes questing for essential sugar sources, oviposition sites and bloodmeal [161]. This technique was first developed by Israel-based researchers [161] and has been currently adopted by various research groups working in Africa, Florida (USA) and Israel [162] for trapping mosquitoes. Most formulations of ATSB involve the use of fruit juices from guavas and mangoes as phytochemical lures, sugar solution as feeding stimulant, and an oral toxin of 1% boric acid that kills mosquitoes upon ingestion [106, 160]. The technique has been successfully deployed for mass trapping of mosquitoes during vector surveillance operations, and for studies aimed at reducing the proportion of endophagic female mosquitoes [163]. Although this new technique is still in the early stages of development, upscaling of its potential to cover large field applications would prove it a powerful malaria vector management tool that complements the existing vector control strategies. For instance, in a field assessment study conducted in Mali, ATSB significantly reduced indoor feeding mosquito populations by 90% suggesting its great potential to control malaria vectors [163]. Additionally, spraying of ATSB on plants was found to reduce the relative abundance of female and male anopheline mosquitoes (by about 90%) with a concomitant reduction in the completion of the gonotrophic cycle [162]. Irrespective of the availability of high-favoured sugar-rich sources, Beier et al. demonstrated that ATSB methods reduced the densities of female anopheline mosquitoes in arid oases during a 47-day field trial study [164]. In summary, these testimonial reports demonstrate the efficacious impact of ATSBs in reducing the prevalence of malaria-transmitting mosquito populations as well as reducing their reproduction cycles.

### Larvicidal agents derived from plants

Mosquitoes spend a considerable amount of time in the water during the development of juvenile stages. Therefore, vector control interventions targeting the larval habitats could considerably suppress the populations of adult mosquitoes consequently contributing to reduced vectorial capacity and parasite transmission [165]. According to WHO [166], larviciding complements the existing vector controls in regions where the sites are “few, fixed and findable” such as urban and rural settings, potentially protecting several households within a small radius. As a component of IVM and larval source management (LSM), the approach reduces the proportion of both indoor and outdoor feeding mosquitoes, hence lowering residual malaria transmission rates [81, 167, 168]. Historically, it is one of the reported successful strategies of mosquito control [26], but its operational implementation in the prevention of malaria in sub-Saharan Africa is limited possibly due to its labour intensiveness, robust technical difficulties, the variability of vector site preferences and demand for frequent applications [169]. Despite these constraints, community-based participatory small scale field trial programmes using formulations of microbial larvicides, *Bacillus thuringensis* var. *israelensis* (*Bti*) and *Bacillus sphaericus* (*Bs*), in African countries such as Gambia, Kenya, Tanzania, Burkina Faso, Côte d’Ivoire, and Benin have shown revitalizing efforts to revive larviciding for malaria vector control [170–177]. Unfortunately, apart from the high cost of these larvicides, the emergence of resistance through larval midgut modifications pose a challenge to their sustainability [178], a scenario that imperatively calls for cost-effective and resistance-resilient chemistries.

Many plant extracts have been investigated for bioactivity against immature stages of mosquito vectors, several with promising efficacies. However, only a few have undergone chemical characterization to elucidate the bioactive ingredients, the core of phytochemistry research that promotes optimisation of plant compounds into vector control [87]. The plant derivatives reported to have larvicidal activity include; N-containing alkaloids, limonoids, phytoecdysteroids, sesquiterpene lactones, flavonoids, essential oils, naphthoisoquinolines, tannins and saponins from *Annonaceae*, *Asteraceae*, *Cyperaceae*, *Ebeneceae*, *Euphorbiaceae, Lamiaceae*, *Lauraceae*, *Leguminosae*, *Meliaceae*, *Cledophoraceae*, *Labiatae*, *Oocystaceae* and *Rutaceae* families [87]. A summary of plant-sourced larvicidal agents has been presented in Table 2. Most of these compounds exert direct toxicity on application to mosquito breeding water, while others cause growth inhibiting effects similar to those exhibited by insect growth regulators (IGRs), reducing survival and development of mosquitoes [87]. For instance, pyridone alkaloids from *Ricinus communis* and sesquiterpene lactones from *Tithonia diversifolia* reduced the survival of *An. gambiae* (*s.s.*) larvae by 60–95% at LC_50_ 0.18 mg/ml and LC_50_ 0.33 mg/ml, respectively [179]. Naphthoisoquinolines from *Lantana viburnoides* and *Plumbago zeylanica* have shown activity against *An. gambiae* (*s.s.*) and *An. arabiensis* larvae [180, 181]. Well-studied mosquito control agents from plants are insect growth regulatory compounds and essential oils.

**Table 2:** A summary of some larvicidal compounds derived from plants

Plant	Active compound	Dosage at LC_50_	Mosquito species	Published source	Mode of action
Non volatiles					
*Ricinus communis*	Pyridone alkaloids	0.18 mg/ml	*An. gambiae* (*s.s.*)	[179]	Toxicity
*Tithonia diversifolia*	Sesquisterpene lactones	0.33 mg/ml	*An. gambiae* (*s.s.*)	[179]	Toxicity
*Plumbago dawei*	Plumbagin, β-sitosterol	4.1 μg/ml	*An. gambiae* (*s.s.*)	[180]	Toxicity
*Azadirachtica indica*	Azadirachtin, salanin, deacetylgedunin	0.014–0.078 ppm	*An. stephensi*	[183]	Toxicity and growth disruption
*Turraea abyssinica*	Mzikonone, 1α-12α- diacetoxy-1,2-dihydro-7-deacetyl-3β-7α-dihydroxyazadiron, 12-α-acetoxy-7-deacetylazadiron	265 ppm	*An. gambiae* (*s.s.*)	[185]	Toxicity
*Turraea cornucopia*	Mzikonone, 1α-12α- diacetoxy-1,2-dihydro-7-deacetyl-3β-7α-dihydroxyazadiron, 12-α-acetoxy-7-deacetylazadiron	202 ppm	*An. gambiae* (*s.s.*)	[185]	Toxicity
*Melia volkensii*	Salannin, volkensin	5.4 mg/l	*An. arabiensis*	[186]	Toxicity
*Dysoxylum malaricum*	3β,24,25-trihydroxycycloartane	2.5–6.5 ppm	*An. stephensi *	[187]	Toxicity and growth disruption
*Dysoxylum beddomei*	Beddomeilactone	2.5–6.5 ppm	*An. stephensi*	[187]	Toxicity and growth disruption
*Vitex payos*	Stigmasterol, 20-hydroxyecdysone, γ-sitosterol	0.25–10 ppm	*An. gambiae* (*s.s*)	[188]	Toxicity and growth disruption
*Vitex schiliebenii*	Stigmasterol, 20-hydroxyecdysone, γ-sitosterol	0.25–10 ppm	*An. gambiae* (*s.s.*)	[188]	Toxicity and growth disruption
*Camellia sinensis* (tea)	Proanthocyanidins	5.52 ppm	*An. gambiae* (*s.s*.), *An. arabiensis*	[189]	Toxicity and growth disruption
Essential oils					
Neem oil	Azadirachtin	11 ppm	*An. gambiae* (*s.s.*)	[198]	Toxicity
*Cryptomeria japonica*	Kau-16-rene, β-elemol	5.55–134.84 μg/ml	*An. gambiae* (*s.s.*)	[199]	Toxicity
*Schinus terebinthifolia*	δ-3-carene	202.15–2,625.20 ppm	*An. gambiae* (*s.s.*), *An. arabiensis*	[200]	Toxicity
*Plectranthus amboinicus*	Thymol, carvacrol	55.20 ppm	*An. gambiae* (*s.s.*)	[201]	Toxicity
*Ocimum canum*	Tannins, phenol, saponin, alkaloid, steroid, flavonoids, triterpenoid	49.51 × 10^−3^ mg/ml	*An. gambiae* (*s.s.*)	[202]	Toxicity
*Cinnamomum osmophloeum*	Trans-cinnamaldehyde	11.91–63.63 μg/ml	*An. gambiae* (*s.s.*)	[204]	Toxicity
*Zanthoxylum armatum*	Monoterpenes	58 ppm	*An. stephensi*	[205]	Toxicity
*Trychyspermum ammi*	Thymol	80 mg/ml	*An. stephensi*	[206]	Toxicity

### Plant-derived insect growth regulators as potential agents for vector control

**Fig. 3:**
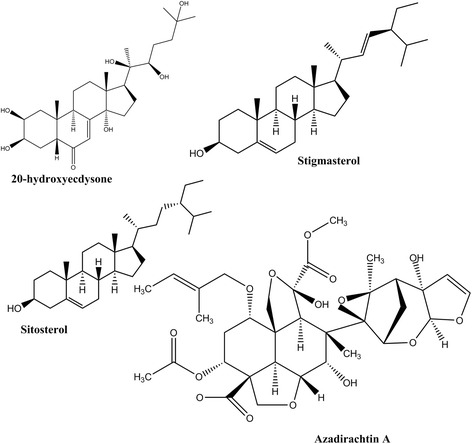
Plant-based insect growth regulators with potential for control of mosquito larvae. The structural similarity of the above compounds with endogenous insect hormones dysregulate normal physiology and development culminating to death or impaired morphology

Limonoids (i.e. sesquiterpenoids and triterpenoids) and phytoecdysteroids (Fig. 3) are plant-based compounds derived from *Azadirachta indica* (neem), *Melia azedarach*, *Vitex payos*, *Vitex schiliebenii*, *Melia volkesnii*, *Plumbago zeylanica*, *P. dawei*, *P. stenophylla*, *Hugonia castaneifolia*, *H. busseana*, *Dysoxylum malabaricum*, *D. beddomei*, *Turraea abyssinica* and *Turraea cornucopia*. These compounds occur in a small portion of 5–10% of terrestrial plants and may show diverse structural characteristics which are associated with efficacy against insect juveniles by mimicking the endogenous developmental hormones [182]. In previous studies conducted by Nathan et al. limonoids from neem and *Melia azedarach* elicited 95–100% mortality at 1 ppm against *An. stephensi* larvae [183, 184]. In another similar study, limonoids sourced from *Turraea abyssinica* and *T. cornucopia* inhibited larval development in *An. gambiae* (*s.s.*) at a range of LC_50_ 202–265 ppm indicating the requirement for much higher concentration to achieve larval inhibition [185]. Triterpenoids (salannin and volkensin) derived from *Melia volkesnii* caused larval mortality at 5.4 mg/l against *An. arabiensis* [186]. Similarly, triterpenes from *Dysoxylum malaricum* and *D. beddomei* caused 90% larval mortality against *An. stephensi* [187]. Moreover, phytoecydsteroids (20-hydroxyecdysone, γ-sitosterol, stigmasterol) sourced from *Vitex payos*, *V. schiliebenii* and *Plumbago spp* have been reported to exert pronounced toxicity (100% mortality) on developing *An. gambiae* (*s.s.*) larvae at relatively high doses and inducing developmental defects at sublethal doses [180, 188]. Recently, Muema et al. reported non-steroidal compounds, proanthocyanidins from green tea leaves, that produced similar IGR-related effects on developing malaria mosquito larvae suggesting their potential control mosquito populations at a low dose of 5 ppm [189]. Additionally, *Agerantum conyzoides*, previously reported to possess anti-juvenile hormone precocenes and bioactivity against *Culex*, *Aedes* and *An. stephensi* was demonstrated to elicit toxicity and inhibit the precocious larval development of *An. gambiae* (*s.s*) and *An. arabiensis* by inducing abnormal larval-pupal intermediates and disrupted adult emergence [190].

### Mechanism of action of plant-derived insect growth regulators

The phytoecdysteroids structurally resemble plant growth steroids (brassinosteroids), but they defend the plants against phytophagous insect attacks [188]. On ingestion, these compounds produce detrimental effects on insect development causing the insect to die as a result of moulting failure [191]. Structural resemblance of these compounds to endogenous insect developmental hormone 20-hydroxyecdysone (20-E) is believed to interfere with moulting process through competition for the same endogenous hormone receptors arresting larval development at larval-pupal intermediates and consequently death [188, 192]. Although the distinct mode of action of phytoecdysteroids and limonoids is ambiguous, these compounds are speculated to either antagonise or agonise insect ecdysteroid and juvenile hormone receptors leading to disruption of endocrine balance [182, 192]. In addition to interfering with the hormonal balance, neem derivatives target cholinergic nerve transmission, hence disrupting neuromuscular coordination [147]. Further, Azadirachtin has been reported to exhibit anti-mitotic effect by disrupting tubulin polymerization [193] and cell cycle arrest by down-regulating cyclin B and D1, in addition to inducing pro-apoptotic signals [194]. On exposure to these compounds, some juveniles exhibit demelanized cuticle, absence or reduced chitin content, extended larval phase, elongated abdominal region, abnormal behaviour changes (tonic immobility) and adults with deformed flight muscles that fail to thrive suggesting dysregulation of neuroendocrine system [195–197]. Besides causing growth regulatory defects, exposure to mosquitoes to these compounds at sublethal doses negatively impact fecundity, reproductive fitness and egg viability - suggesting the potential of these compounds in insect control [147].

### Use of plant-derived essential oils in larval control

Manipulation of larval habitats with biodegradable plant-derived essential oils has replaced the use of kerosene for mosquito control [198]. These complex blends form a thin emulsion layer over the surface of mosquito breeding water, hence preventing oxygen entry, reducing amounts of water-dissolved oxygen, and induces larval intoxication upon ingestion. Existing reports indicate that essential oils evoke larval toxicity and are potent against *An. gambiae* (*s.s.*), *An. arabiensis* and *An. stephensi* [199] (Table 2). Findings by Kweka et al. showed that δ-3-carene from *Schinus terebinthifolia* essential oil was responsible for larval mortality rates of 13.75–97.91% in *An. gambiae* (*s.s.*) [200]. Thymol and carvacrol from *Plecranthus amboinicus* produced lethality at LC_50_ 55.20 ppm and LC_90_ 99.09 ppm against *An. gambiae* (*s.s.*) within 24 h of exposure [201]. Babatunde et al. reported that *Ocimum canum* essential oil reduced survivorship of *An. gambiae* (*s.s.*) larvae at LC_50_ 49.51 × 10^−3^ mg/ml and LC_90_ 103 × 10^-3^ mg/ml [202]. It has also been reported that essential oils of *Plectaranthus glangulosus* and *Callestemon rigidus* were active against *An. gambiae* (*s.s.*) at LC_50_ 7.37 ppm and 99.61 ppm respectively [203]. In addition, laboratory- and field-based studies performed using *An. gambiae* (*s.s.*) demonstrated that trans-cinnamaldehyde from *Cinnamomum osmophloeum* and kau-16-rene, β-elemol from *Cryptomeria japonica* leaf essential oils caused larval toxicity at LC_50_ 11.91–63.63 μg/ml and LC_50_ 5.55–134.84 μg/ml, respectively [199, 204]. Moreover, monoterpenes from *Zanthoxylum armatum* essential oil evoked lethality to *An. stephensi* at LC_50_ 58 ppm [205], while thymol from *Trychyspermum ammi* caused larval mortality at LC_50_ 80 mg/ml [206]. Neem oil formulation killed late larval instars of *An. gambiae* (*s.s.*) at LC_50_ 11 ppm and inhibited adult emergence at IC_50_ 6 ppm [198].

It is important to note that the efficacy of different plant compounds depends on various parameters such as; extraction method, geographical location of the plant, plant part used, concentration, test mosquito species, solvents used, the photosensitivity of some phytochemicals and time of extraction [87].

### Synergistic phenomena in malaria control

Incorporation of plant-based compounds to potentiate the effects of the existing vector control methods has been studied, and findings from these studies indicate the synergistic potential of botanical derivatives in reducing risks of malaria transmission. For instance, in a randomized trial study carried out in the Bolivian Amazon, a reduction of 80% in malaria episodes was reported among participants who used plant-based repellent (PMD) and slept under LLINs, thus indicating a synergistic potential of PMD to LLINs relative to placebo group which slept under LLINs only without using the repellent [207]. Additionally, in a community-based clustered randomised trial, Deressa et al. reported that the combined use of LLINs and mosquito repellent (Buzz-Off® petroleum jelly, essential oil blend) significantly reduced malaria infection by 34–47% relative to the control groups which received LLINs alone [208]. Kweka et al. showed that individual compounds and their blends of plant-derived menthol propylene glycol carbonate (MR08) and DEET offered a protective efficacy in the range of 92–100%, suggesting that these blends could be of additional value for personal protection in the absence of IRS and LLINs [209]. Elsewhere, Stewart et al. found out that indoor application of ATSBs in combination with LLINs could be a promising strategy to control pyrethroid-resistant mosquitoes [210].

### Regulatory issues, advancements and commercialization of plant-derived mosquito control compounds

The demand for relatively safe and effective plant-based mosquito control agents by consumers continues to increase relative to synthetic counterparts [86]. However, despite the extensive research on the insecticidal potential of plant-based compounds, only a few have been successfully registered and approved for commercialization [211]. Strict regulatory laws imposed on the marketing of these compounds in many developed countries have slowed down the full exploitation of many established effective plant-derived chemistries to control nuisance insects. The concerns on potential risks associated with plant-derived compounds to environment, humans and non-target organisms are variable and majority lack of experimental basis [89]. In spite of these issues, EcoSMART Technologies Inc. (Atlanta, USA) has succeeded in introducing plant-based insecticidal compounds for agricultural and consumer utilisation [89]. In developing countries rich in biodiversity, many promising plant-derived compounds remain untapped for control of harmful insects, although in some cases, whole plant parts or crude extracts have been reported to protect humans from mosquito bites effectively [144]. Concerns raised on the variability of product chemistries during pre- and post-harvest processing, that may affect the activity of the end product, are subject to debate [87, 95]. Some compounds are effective when in a cocktail of other plant components, whereas others require being isolated and purified. Nevertheless, whether to use crude extracts, for instance, essential oil to drive away mosquitoes or a formulation of the major chemical components is subject to user preference [95]. Stability of the isolated compounds under different environmental conditions must be considered because some compounds may change the chemical conformation of functional groups upon storage due to photosensitivity and other environmental factors [89]. For instance, neem derivatives and pyrethrum compounds are highly sensitive to UV exposure leading to degradation that in turn reduces their efficacy [89].

Residual efficacy of plant-based repellents and ATSBs is also under consideration. While many repellent compounds offer high protection efficacy similar to DEET against malaria mosquito vectors, volatility minimises their longevity to mediate protection [86]. Advancements in technologies such as encapsulation and microencapsulations, nanoemulsions and fixatives have been pursued to improve the longevity of potent repellent compounds [86, 212]. For instance, slow-release encapsulated citronella oil nanoemulsion has been used to increase the efficacy of citronella-treated fabrics for up to 30 days [213]. The shelf life and efficacy of neem-based products have been enhanced through microencapsulation, microemulsions, inclusion complexes and granular formulations [214, 215]. Membrane-based ATSBs enhance the release of bio-lures and increasing their efficiency in field applications (unpublished information from Günter Müller, Hebrew University, Jerusalem, Israel). Although enhancement of longevity would mean prolonged protection against mosquitoes, potential issues regarding environmental toxicity may arise. Environmental impact of many plant compounds is generally considered low owing to their biodegradability and short half-lives of < 30 h [89]. Neem derivatives have fewer impacts on pollinators, natural pest predators and vertebrate species, despite its efficacy in controlling more than 300 insect species [216, 217]. In contrast to synthetic insecticides, many plant-derived compounds exhibit no persistence, bioaccumulation and biomagnification [89]. Thus, it would be more advantageous when persistence is reduced to minimise negative implications. Widespread application of plant-based compounds especially mosquito repellents creates wary on the selectivity of these compounds to target invertebrates [95]. Though generally regarded safe, it is yet to be established if some of these repellents could have negative impacts on non-target arthropods.

Other drawbacks to the commercialization of plant-derived mosquito control agents involve sustainability of the botanical resource, industrial confidence in the products, up-scaling potential of plant resource products, standardisation of chemically complex extracts, slow action of other compounds and availability of competing products such as newer synthetics, fermentation and microbial products.

### Future perspectives

As the world’s human population continues to increase (world’s population growth rate currently approximated at 1.24% annually [218]), more land is needed to sustain developments, agriculture and settlements. These anthropogenic activities contribute to opening up of potential mosquito breeding sites and even speciation of malaria vectors [172, 219]. Also, the overwhelming development of vector resistance to the currently available synthetic insecticides, following persistent application, continues to challenge the effective control towards malaria transmission [8, 220]. It is therefore anticipated that the identification of bioactive plant compounds will continue for improved management of malaria-transmitting mosquito vectors. A handful of biologically active compounds identified from plants is yet to be exploited for controlling insect pests and vectors on a large scale. Therefore, ‘bench to field’ transition of laboratory tested bioactive compounds and subsequent incorporation into IVM could offset insecticide resistance, undoubtedly reducing malaria vector populations and risk of malaria transmission by greater magnitudes. Successful interventions to control malaria-transmitting mosquitoes using plant-derived compounds would require available, scalable and sustainable technologies for both local and large-scale manufacture. Nanotechnology is currently revolutionising the production of market pesticides. Production of plant-derived nanoparticles and nanoencapsulation compounds increases the longevity of essential oils through slow-release phenomenon conferring prolonged protection against mosquito bites [86]. Furthermore, the silver nanoparticle larvicidal agents derived from plant extracts will find useful applications in larviciding [221]. Advances in applied nanobiotechnology have revolutionised synthesis of plant-based silver nanoparticles that are currently being reported effective against even insecticide resistant disease-transmitting vectors, primarily targeting the immature stages at low dosages of 1–30 ppm [222]. The membrane-based ‘attract and kill’ systems will also in future be revolutionising field-based applications of bio-lure compounds for mass trapping of mosquitoes (unpublished information from Günter Müller, Hebrew University, Jerusalem, Israel). It is therefore anticipated that more robust technologies for controlling malaria vectors using bio-products are underway. Plant-derived compounds could potentially be used for controlling mosquito vectors by manipulating their behaviour and possibly replace pyrethroids in impregnating bed nets as suggested by Deletre et al. [141].

## Conclusion

Comprehensive understanding of the mechanistic role of mosquito olfaction, odour coding and larval ecology is crucial for developing new strategies for disrupting malaria transmission cycle. Integrated vector management programmes advocate for strategies that aim at improving the cost-effectiveness, efficacy, ecological soundness and sustainability of control interventions. In nature, economically feasible plant bioactive compounds are in abundance, many of which are unexploited for vector control. We state that the tools and compounds presented in this review, despite showing promising efficacy against outdoor mosquito populations under laboratory and small field trials, effectiveness under large-scale field trials and various epidemiological settings remain unexplored. The blends of secondary metabolites extracted from plants cannot be used alone, hence will require being integrated with the existing vector control methods so as to provide synergistic tools that can sustainably help to reduce and possibly eliminate malaria vector populations.

## Abbreviations

ATSB: Attractive toxic sugar bait
DEET: N,N-diethyl-3-methylbenzamide
EIRs: Entomological inoculation rates
GABA: Gamma aminobutyric acid
IRS: Indoor residual spraying
IVM: Integrated vector management
LLINs: Long-lasting insecticide treated nets
OBPs: Odorant-binding proteins
ORs: Odorant receptors
PMD: *p*-methane-3,8-diol
WHO: World Health Organization
